# *Lachancea quebecensis* a Novel Isolate for the Production of Craft Beer

**DOI:** 10.3390/foods12183347

**Published:** 2023-09-07

**Authors:** Valeria Galaz, Wendy Franco

**Affiliations:** 1 Department of Chemical Engineering and Bioprocess, Pontificia Universidad Católica de Chile, Ave. Vicuña Mackenna 4860, Macul, Santiago 7820436, Chile; vgalaz@uc.cl; 2 Departamento de Ciencias de la Salud, Carrera de Nutrición y Dietética, Pontificia Universidad Católica de Chile, Ave. Vicuña Mackenna 4860, Macul, Santiago 7820436, Chile

**Keywords:** insects, yeasts, *Lachancea*, beer

## Abstract

Yeasts are ubiquitously present in different natural sources. Some of these yeasts have interesting characteristics for the production of fermented food products. This study characterized *Lachancea thermotolerans* and *L. quebecensis* isolated from insects to determine their brewing potential. The yeasts were evaluated according to their fermentative potential in glucose and maltose-defined media and their resistance to ethanol and hop. Finally, craft beer was elaborated at a laboratory scale (10 L). The yeasts utilized glucose as the only carbon source and produced 3.25 ± 1.77, and 4.25 ± 1.06% (*v*/*v*), of ethanol for *L. thermotolerans* and *quebecensis*, respectively. While in the maltose-defined medium, ethanol content reached 3.25 ± 0.45, and 3.92 ± 0.36, respectively. The presence of alpha acids and ethanol affected the growth of *L. quebecensis*, which showed lower growth at 90 IBU and 8 ethanol% (*v*/*v*) mixtures. The craft beer brewed with *L. quebecensis* in monoculture experiments showed fruity flavors associated with ethyl acetate and isoamyl acetate. The ethanol content reached 3.50 ± 0.46% (*v*/*v*). The beer pH was 4.06 ± 0.20, with a lactic acid concentration of 1.21 ± 0.05 g/L. The sensory panel identified the beer as “fruity”, “floral”, “hoppy”, “sweet”, and “sour”. To our knowledge, this is the first time *L. quebecensis* was reported as a potential candidate for sour beer production with reduced ethanol content.

## 1. Introduction

*Saccharomyces* species are traditionally used to produce alcoholic beverages, including beer. Domestication of the yeast has led to a specialization to produce high ethanol yields rapidly, allowing standardization of the final product. However, in recent years, emerging lifestyle trends have shifted the beer industry towards craft breweries where diverse and original styles can be made. To achieve this, yeast species other than *Saccharomyces* have been used and studied for beer production. Non-*Saccharomyces* yeasts are less efficient alcohol producers but a source of other metabolic products that enhance fermented foods’ flavor, aroma, and texture [1]. For example, they have been previously used in the wine industry to produce wines with enhanced mouthfeel and aroma [2,3]. And in the last years, they have also been used to produce reduced-alcohol beers [4,5] and enhance the beer’s aroma [6,7].

The non-*Saccharomyces* yeasts used in the food industry are commonly isolated from natural sources, the vineyard and wineries environments the most common [1,8,9,10]. However, yeast can be isolated from different natural sources, including insects. Insects represent an interesting habitat for searching for novel yeasts valuable to the food industry. Furthermore, given the host, yeasts isolated from insects might have characteristics that make them unique, such as resistance to stress conditions and diverse carbohydrate metabolism. This last trait is particularly interesting in beer making since wort comprises different carbon molecules available for yeast utilization.

Madden et al. (2018) studied the yeast diversity in wasps and bees and isolated 64 yeasts distributed in the genus *Candida*, *Hanseniaspora*, *Lachancea*, *Metschnikowia*, *Pichia*, and *Saccharomyces* [11]. Among the isolated yeasts selected, *L. thermotolerans* and *L. fermentati* isolates were able to utilize maltose as a carbon source, suggesting its potential as starters for beer production.

Species from the *Lachancea* genus have been previously studied for beer production. Zadaniewicz et al. (2020) reported that *L. thermotolerans* MN477031 was able to produce beers of about 4.25 ± 0.17 to 4.3 ± 0.02 (% *v*/*v*) ethanol in Lubeski and Marynka worts, respectively. A commercial *Lachancea* strain (Concerto) was able to produce a beer with a 3.82 ± 0.06 (% *v*/*v*) alcohol content [12]. Bellut et al. (2019) reported that *L. fermentati* can produce beer with an even lower ethanol content (2.21 ± 0.17% *v*/*v*) [13].

The genus *Lachancea* is also characterized by its ability to produce significant lactic acid concentrations and alcoholic fermentation [14]. This attribute has been explored to produce enhanced volatile acidity in wines [15] and sour beer [16,17,18].

The demand for sour beer has recently increased, with a market growth of about 43% in 2019 [19]. This beer type is commonly produced by spontaneous fermentation during long-term storage in wood barrels, in which organic acids are produced by the microbial consortium, resulting in beers with low pH [20]. However, some studies have evaluated the potential of introducing starter cultures to drive organic acids production, particularly lactic acid production. This has been accomplished using lactic acid bacteria (LAB) as starter cultures [21]. However, since yeasts, such as *Lachancea* spp., can produce organic acids, they might be an alternative to produce sour beer.

*L. thermotolerans* possesses the capacity to produce lactic acid through heterofermentative metabolism, contributing to the characteristic tartness and acidity desired in sour beers [18]. Its compatibility with *Saccharomyces cerevisiae* in co-fermentation scenarios has been explored to create a complex flavor profile that includes sour and ester-driven aromatic attributes [22]. The use of yeasts to produce sour beers is a relatively new research topic and has been reported as a LAB-free method [21]. Its utilization not only diversifies the microbial consortium of sour beer fermentation but also introduces the prospect of achieving consistent and repeatable souring effects. As research and brewing practices evolve, the application of *L. thermotolerans* and other species, such as *L. fermentati*, in sour beer production continues to unveil new dimensions of flavor, aroma, and fermentation possibilities, enriching the landscape of contemporary craft brewing [12,13,16,23].

This study aimed to isolate, select, and evaluate the brewing potential of selected *Lachancea* spp. isolated from different insects. Four insect samples were collected at the San Francisco vegetable patch and the Entomology laboratory located at the Pontifical Catholic University of Chile (Santiago, Chile). Isolated yeasts were identified and later analyzed to determine their fermentative profile, resistance to ethanol and hop. Two selected *Lachancea* isolates were then used to brew Pale Ale Craft beer.

## 2. Materials and Methods

### 2.1. Insects

The yeast strains isolated and discussed in this research were obtained from four insect samples: ladybug (*Coccinelliade*) and cabbage worm (*Leptophobia aripa boisduva*) collected from the San Francisco vegetable patch located at the Pontifical Catholic University of Chile. While corn (*Sitophilus zeamais*), and wheat flour (*Tribolium castaneum*) weevils were provided by the Entomology Laboratory at the same University).

### 2.2. Yeasts Isolation and Identification

#### 2.2.1. Isolation

Following the methodology reported by Nguyen et al. (2008), the collected insects were placed individually in plastic containers with lids, previously sanitized with 70% ethanol, and lined with a paper towel moistened with sterile distilled water. The containers were kept at room temperature (25 ± 2 °C) for three days. After this time, it was verified that the insects were dead, and then they were submerged in 95% ethanol for 150 s for surface disinfection. Clean insects were washed with sterile distilled water and gently vortexed (VX-200 vortex mixer, Labnet, Edison, NJ, USA). Wash water was used as a negative control [24]. Each disinfected insect was transferred to an Eppendorf tube with 900 μL of 1% peptone water (Buffered Peptone Water, OXOID CM0509, Basingstoke, Hampshire, UK) and ground using a small, sterilized spatula until disintegrated. The supernatant was separated from the particulate and was serially diluted with peptone water [25]. Different dilutions were plated in Yeast Glucose Chloramphenicol Agar (YGC, Oxoid, London, UK) and incubated at 25 °C for 72 to 96 h. Observed colonies were classified according to their morphology. Three clones from each morphology type were isolated and stored for further identification.

#### 2.2.2. Identification

Pure colonies from each culture plate were seeded in Sabouraud Broth (SBB, Merck; Darmstadt, Germany) and then incubated for 24 h at 25 °C until reaching an approximate 10^6^ CFU/mL concentration.

DNA was extracted using the GeneJET Genomic DNA Purification Kit (Thermo Scientific, #K0722, San Louis, MI, USA), following the manufacturer’s instructions. Then, the extracted DNA was amplified in PCR using the reagent mixture made up of 2X Master Mix (Bio-Rad, Hercules, CA, USA), chromosomal DNA, nuclease-free water (Thermo Scientific, #R0581, San Louis, MI, USA), and two primers: NL-1 (5′-GCCATATCAA TAAGCGGAGGAAAAG-3′ reverse) and NL-4 (5′-GGTCCGTGTTTCAAGA CGG-3′ forward) [26]. The amplifications obtained were sent to Macrogen (Gangnam-gu, Seoul, Republic of Korea) to be sequenced.

To identify the isolated yeasts, the sequences obtained were analyzed using the Basic Local Alignment Search Tool (BLAST 2.2.26) algorithm [27] accessible in GenBank, using the non-redundant nucleotide database. Only alignments greater than 95% coincidence in identity were considered for identification purposes.

Selected yeast isolates were then characterized to discriminate the potential brewing isolates. 

### 2.3. Fermentative Profile in Defined Medium

The ability of the yeasts to produce ethanol from sugars commonly found in wort was determined in glucose and maltose-defined media.

#### 2.3.1. Glucose Defined Medium

Seven grams of Yeast Extract (YE, Oxiod LP0021, London, UK) were diluted in 1000 mL of distilled water as a nutrient source for the yeasts to be inoculated, and the mixture was sterilized. Then, the medium was allowed to cool to 60 °C, where 180 g of D-(+)Glucose (Merck 1.08342.1000, Rahway, NJ, USA) was added. 

#### 2.3.2. Maltose Defined Medium

This medium was prepared by mixing 100 g of malt extract (Oxoid LP0039, London, UK) and 10 g of servomyces (Lallemand, Montreakl, QC, Canada) in 1000 mL of distilled water, and the mixture was sterilized. Servomyces were used as a nutrient source for the yeasts to be inoculated.

The fermentations were carried out in two-mouth sterile flasks adapted with airlocks to allow the exit of carbon dioxide and prevent the entry of oxygen and thus maintain anaerobic conditions. Culture media portioned in triplicate were inoculated (1%) with fresh yeast culture (10^6^ CFU/mL) and incubated at 27 °C and 150 rpm for six days. Aliquots were collected on days 0, 1, 2, 3, 5, and 6. On each sample, the following was determined: concentration of soluble solids (°Plato) using a refractometer (pocket refractometer pal-1 Atago, Tokyio, Japan), pH with a pH meter (pL 700PV, Meter Toledo, Columbus, OH, USA), and cell count by plating on YMA agar (Oxoid).

Ethanol concentrations were measured by High-Pressure Liquid Chromatography (HPLC) using a 30 cm HPX-87H column (Bio-Rad Laboratories, Hercules, CA, USA) for component separation [28]. The column temperature was maintained at 37 °C; component samples were eluted with 0.03 N sulfuric acid at a 0.6 mL/min flow rate. A Thermo Separations UV6000 diode array detector (Spectra System Thermo Scientific, Waltham, MA, USA) and a Waters model 410 refractive index detector (Waters Corp., Millipore Corp., Billerica, MA, USA) connected in series with the detector diode array were used to determine the metabolite. External standardization of the detector was performed using four concentrations of the standard compound. Maltose was determined using the Maltose Assay Kit (Merck, Rahway, NJ, USA) following the manufacturer’s protocol.

#### 2.3.3. Resistance to Hop and Ethanol

The methodology Michel et al. (2016) reported was used to determine the resistance of the selected isolates to hop and ethanol. Briefly, wort extract (Patagonian Malt, Patagonia, Chile) was mixed with deionized water. The pH was adjusted with NaOH 10 M. The mixture was sterilized at 100 °C for 45 min [29].

The base wort was supplemented with 6% iso-alpha-isomerized acid (Mundocervecero, Santiago, Chile) to determine hop resistance thresholds until reaching 50 and 90 International Bitterness Units (IBU). For ethanol resistance, ethanol (96% *v*/*v*) was added to adjust the concentration to 5% and 8% (*v*/*v*). For the combined resistance test, each standardized must with iso-alpha isomerized acid (50 and 90 IBU) was added with ethanol (96% *v*/*v*) until reaching concentrations of 5 and 8% (*v*/*v*).

One mL of each type of wort was transferred to Eppendorf tubes, and these were inoculated with fresh cultures of yeast isolates at a concentration of 10^6^ CFU/mL. Two hundred uL of each mixture were transferred to a 96-well microtiter plate (Costar, New York, NY, USA). The plate was sealed with permeable plastic and placed inside a photometer (Infinite F200 Pro Tecan, Grödig, Austria) at 25 °C. Every 10 min, the optical density was measured, followed by 8 min of strong orbital shaking. Non-inoculated base wort and musts inoculated with English yeast S04 (Lallemand) were used as a negative and positive control, respectively.

### 2.4. Craft Beer Production at Laboratory Scale (10 L)

#### 2.4.1. Propagation

The propagation of the yeasts was done following the methodology reported by Bellut et al. (2019). Briefly, a wort consisting of 75 g/L malts and 30 g/L of glucose was sterilized at 121 °C for 15 min. 150 mL was placed in double neck Erlenmeyer flasks provided with airlocks. Single yeasts cultures taken from YMA plates were inoculated in the wort. The mix was left for fermentation for 48 h at 25 °C and 100 rpm. After this time, samples were collected and plated in YMA to verify viability [13].

#### 2.4.2. Beer Making

Whole grain barley was mixed with water and left to macerate for 60 min at 60 °C. Every ten min, a manual homogenization was carried out. After this, the macerated grains were washed with water at 75 °C. Then the wort was boiled for 60 min. During this process, hop (Mundo Cervecero, Santiago, Chile) was added in a cascade sequence. First bitter hop was added at the beginning of the boiling process. Fifteen min before the boiling step was finished, flavor hop was added, and then after 3 min, aroma hop was added. The mix was transferred to a cold bath and left to cold until 25 °C was reached. Hot trub and hop residue were removed. The pre-activated yeast cultures were inoculated (10^6^ CFU/mL), and the mix was left for fermentation and 25 °C for ten days. After this time, the result beers were bottled in 350 mL amber bottles. Six g/L of dextrose were added before closing. The bottles were left for secondary fermentation for seven days at 20 ± 2 °C.

Samples were taken to determine color and bitterness were determined following the methodologies of the European Beer Convention (EBC), pH was measured with a pHmeter (Meter Toledo), and °Plato with a densimeter (Mundo cervecero). Free amino nitrogen (FAN) was determined following the ninhydrin colorimetric method proposed by the European Brewery Convention. Changes in absorbance were determined using a UV-Vis spectrophotometer (UV-M51, UV/VIS, Monza, Italy) at 570 nm [30].

In addition, the concentration of ethanol, lactic acid, glucose, and maltose was determined by HPLC. Volatile characterization was done by solid phase microextraction (SPME-HS, GC 2010 Plus, Schimazu, Kyoto, Japan) and subsequent injection into a gas chromatograph coupled to a mass detector (GCMS, QP 2020). Each chromatogram was analyzed by comparing the mass spectra with those of the NIST-EPA-NIH library of 130,000 spectra. The compounds were determined: Acetaldehyde, Dimethyl sulfur (DMS), Ethyl acetate, 1-propanol, Isobutanol, Isoamyl acetate, and Isoamyl alcohol.

### 2.5. Sensory Analysis

Sensory analysis was carried out following the Beer Judge Certification Program (BJCP) sensory profile analysis procedures. The panel members (8 trained judges) were first instructed to freely associate the beer samples with a beer type (e.g., ale, wheat, Kölsch, Alt, stout, Berliner Weisse, porter, lager; Bock, Märzen, Rauch, Schwarz, Dunkles, IPA, malt beer), followed by an examination of the beer samples according to the BJCP procedure [31]. Next, each panelist was instructed to comment on the different attributes (appearance, aroma, flavor, and mouthfeel). Then, a descriptive analysis was performed to score distinct aroma and taste attributes using a 9 points scale. Lastly, the general impression of the beer tasted was asked.

### 2.6. Statistical Analysis

The results were expressed as a mean ± standard deviation of the mean (SD), with samples in triplicate and two independent runs. For statistical analysis, the Statgraphics Centurion XVI software was used. An analysis of variance (ANOVA) was applied to datasets at a significance level of *p* < 0.05. Lastly, the LSD test was used for multiple data comparisons to establish significant differences with a 95% confidence level.

## 3. Results and Discussion

### 3.1. Yeasts Isolation and Identification

**Figure 1:**
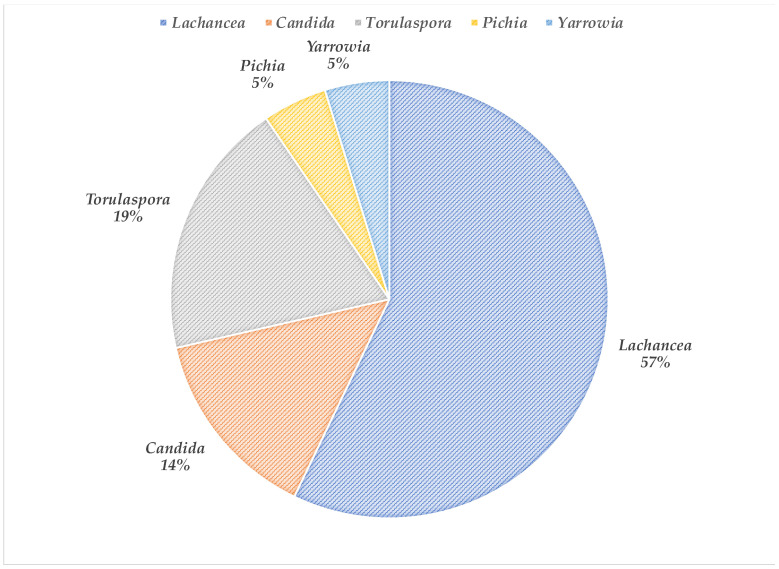
Yeast genus isolated from different insects.

Insect samples were collected at the San Francisco Garden Patch and the Entomology Laboratory at the Pontifical Catholic University of Chile (Santiago, Chile). The insects were prepared, and their extrudates were plated in selective media to isolate yeasts. The culture plates showed different colonies with distinctive morphology, which suggested the presence of different types of yeasts. From this first classification, three clones of each morphology were purified and later used for identification based on *26S* rRNA. Five genera were identified (Figure 1), represented by *Lachancea* (57%), *Torulaspora* (19%), *Candida* (14%), and *Pichia y Yarrowia* (5% each). The most abundantly isolated yeast was identified as *L. thermotolerans*, found in all insect samples, followed by *T. delbrueckii* isolated from corn and wheat flour weevil samples. *Candida boidinii* was isolated from weevil samples. Finally, a *Pichia guillermondii* was isolated from the wheat flour weevil, and a *Yarrowia* sp. from the corn weevil sample (Table 1).

In our bibliographical research, it was not possible to find publications that reported the microbiological diversity of the insects studied in this work. However, the diversity of yeast species has been reported in other types of insects belonging to the orders of Coleoptera, Lepidoptera, and Hemiptera [24,25,32]. Highlighting the presence of species of *Candida* and *Pichia* isolates. On the other hand, certain species of *Lachancea* have been isolated from insects, particularly from their guts. Having symbiotic relationships with insects, these yeasts play crucial roles in digestion and nutrition. They are often involved in the fermentation of ingested plant material, helping insects break down complex carbohydrates and obtain nutrients from their diets [33].

One well-known example of a *Lachancea* species isolated from insects is *Lachancea thermotolerans*. This yeast species has been found in the gut of fruit flies (*Drosophila* spp.), such as the olive fruit fly [34]. *L. thermotolerans* is known for its ability to ferment various sugars and tolerate high temperatures. Therefore, it has been studied for potential applications in winemaking [1,22,35,36] and beer [12,16,17].

Other less studied *Lachancea* species have also been isolated from insects, such as *Lachancea fermentati* and *Lachancea dasiensis*. These yeasts have been found in the gut of beetles and ants, respectively, and their metabolic capabilities and ecological roles are currently being investigated. In our research, we isolated a novel *Lachancea* specie, *L. quebecensis*. The yeast has been previously isolated from various parts of plants, such as leaves, bark, and flowers [37,38]. Moreover, it has been proposed that these yeasts could be associated with insects, perhaps *Drosophilids*, frequently visiting sap flows and tree bark [39].

The *Lachance* genus was proposed by Kurtzman in 2003 to accommodate a group from several different genera showing similarities at the *rRNA* level [40]. In that sense, *L. quebecensis* has similar morphology and physiological characteristics to *L. thermotolerans* and might have similar capabilities for developing fermented food beverages, including beer.

Non-*Saccharomyces* yeast are known as poor ethanol producers and, therefore, a suitable candidate for the production of reduced or low ethanol content beers [5,7,41,42]. The most studied non-*Saccharomyces* yeasts are *L. thermotolerans* and *T. delbrueckii* [12]. An interesting trait of *Lachancea* species is that they can produce lactic acid along with ethanol formation, which is an attractive capability for bacteria-free sour beer production [20,21]. Given this, our study focused on characterizing selected *Lachancea* isolates to determine their brewing potential.

### 3.2. Screening for the Brewing Potential of Selected Lachance Isolates

#### Glucose and Maltose Defined Media

First, the ability of the isolates to utilize wort sugars was determined in a defined medium made with glucose or maltose as the sole carbon source. Ethanol yield was determined after fermentation of 7 days at 25 °C (Table 2).

All the isolates were capable of producing ethanol from glucose but in different concentrations and yields (g of ethanol produced/g of glucose used). To determine the ethanol capacity for the studied yeasts, they were grouped into four categories, taking the control experiment, made with a commercial *S. cerevisiae* starter, as the standard. With this, four categories were observed: yeasts with similar yield (0.40 to 0.50), moderate yield (0.30 to 0.39), low yield (0.20 to 0.29), and very low yield (0.10 to 0.19). Generally, non-conventional yeasts have low fermentation yields because they are not domesticated. They are more inefficient in producing the metabolite and are inhibited either by the lack of remaining glucose or by ethanol concentrations that exceed their tolerance limit [43]. In our study, the yeasts reached concentrations greater than 10^7^ CFU/mL (Table 2), suggesting that they are not inhibited by the concentrations of ethanol produced but prefer using glucose to increase biomass. However, Contreras et al. (2014) reported that ethanol production and resistance is strain specific in native yeasts [44].

Significant changes in pH were observed during glucose fermentation. The defined medium started with a pH of 6.18 to 6.79, and after seven days of fermentation, values between 3.6 to 4.5 were observed. (Table 2). The reduction in the pH level may be associated with the production of other acidifying metabolites, such as organic acids [16,37,45,46,47].

Considering the ability to use glucose and produce ethanol, four isolates were selected for further studies. Each isolate represented one of the categories (low, medium, and high) used in this study (Table 3).

The selected yeasts were cultivated in a medium rich in maltose (100 g/L) at 25 °C under anaerobic conditions for seven days. All selected yeast strains could assimilate maltose, reflected in the increase in the cell population (approximately 2 log) and ethanol production. Depending on the yeast, the ethanol produced ranged from 3.25 ± 0.45% to 5.67 ± 0.82% *v*/*v*). Interestingly, the *L. quebecensis* isolate had the lowest ethanol production, with an ethanol content of 4.25% (*v*/*v*).

Given that the study aimed to identify potential yeast starters for the production of reduced alcohol beer, two low ethanol producers yeasts [*Lachancea quebcensis* (OP923903) and *L. themotolerans* (OP923897)] were selected for further characterization (hop and ethanol resistance) and craft beer elaboration.

### 3.3. Resistance to Hop and Ethanol

The yeasts used in the brewing industry, in addition to the ability to grow and produce ethanol using the sugars in the wort, must withstand different concentrations of hops and ethanol. The former is added as an ingredient in the hot brewing process, specifically in boiling. This ingredient, which comes from the hop flower, gives bitterness, flavor, and aroma to the wort, depending on the boiling time it is added. It also serves as a natural preserve without causing changes in pH. Therefore, the behavior of the yeast in different hops concentrations must be studied to prevent the process from being negatively affected, for example, the production of stagnant fermentations where unwanted aromas and flavors are formed.

On the other hand, ethanol is a product of the yeast’s metabolism. Therefore, each yeast has a tolerance threshold to ethanol; after this threshold, the microbial growth stops, and the yeast is inhibited, so the fermentation process (substrate use and ethanol production) stops. Given that the yeasts must remain active until the must reach certain physicochemical parameters, such as having a pH below 4.6, an ethanol concentration of approximately 5% (*v*/*v*), and a residual °Plato of 6, it is important to determine the thresholds of the wild yeasts isolated in this study, before determining if they have potential use in the production of craft beers.

To evaluate this, the two selected yeast isolates were inoculated (10^6^ CFU/mL in microplates with a culture medium at two concentrations for ethanol (5 and 8%) and hop (50 and 90 IBU) and a mixture of ethanol/hop. The selected ethanol concentrations correspond to values associated with pale ale-type craft beers up to IPA-type beers. On the other hand, the IBU values studied are related to different types of hoppy beer.

**Figure 2:**
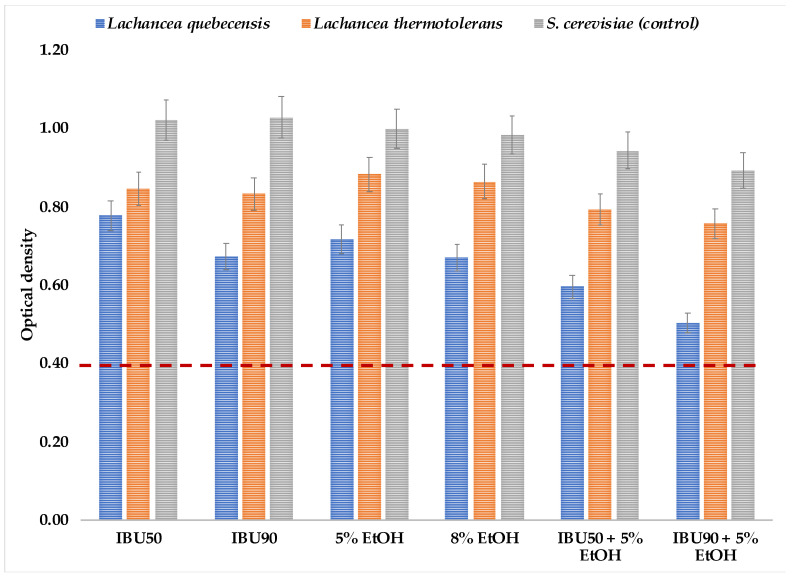
Hop and ethanol resistance of the selected *Lachancea* isolates. Bars represent the optical density after 60 h of incubation at 27 °C. The dashed line indicates the critical value that was used to determine resistance. Values lower than 0.4 were taken as with low or no resistance.

As shown in Figure 2, the isolates increased their optical density. They exceeded the critical value (optical density 0.4) in the medium added with iso-α-acids (IBU) at the two concentrations studied. IBU concentrations did not significantly affect the growth of *L. thermotolerans* which showed a similar behavior at both values. On the other hand, *L. quebecensis* showed more sensitivity to higher IBU. Michel et al. (2016) showed that as the IBU concentration increases, the ability of yeasts to grow is reduced. However, *Lachance* strains have been previously reported to resist significant IBU concentrations. Domizio et al. (2016) reported that some *L. thermotolerant* strains can support at least 60 IBU [16]. Strains of *L. fermentati*, isolated from Kombucha, were also reported as able to resist up to 100 IBU [13,23].

Similar behavior was observed regarding ethanol’s effect on cell growth. Both yeasts increased cell density at both ethanol concentrations in time, with little influence associated with the ethanol content (Figure 2). Still, as expected and in the same way as in the hop resistance study, cell growth is decreased by increasing the ethanol concentration. Non-*Saccharomyces* yeasts tolerate lower ethanol concentrations than *Saccharomyces* species. In winemaking, for example, as the ethanol concentration increases, the natural microflora composed mainly of non-*Saccharomyces* yeasts decreases [48]. However, yeasts such as *L. thermotolerans* could persist after fermentations that reached more than 10% (*v*/*v*) ethanol [49]. On the other hand, *L. fermentati* KBI isolates, studied by Bellut et al. (2020), were resistant to ethanol concentrations up to 7.5% (*v*/*v*) [23].

The mixtures of IBU and ethanol did not significantly affect the growth of *L. thermotolerans*. In contrast, *L. quebecensis* was affected in both combinations, showing lower optical density values, especially for the mixture with 90 IBU, at which the yeast barely increased cell density.

### 3.4. Pale Ale Craft Beer Production

The two *Lachancea* isolates were used to produce Pale Ale craft beer at a laboratory scale (10 L). Table 4 shows the beer’s physicochemical characteristics. Initial wort composition showed an original extract of 12.92% (*w*/*w*), 185.79 Free Amino Nitrogen (FAN), 13.05 °Plato, and a pH of 5.55. The wort had 11.89 g/L of glucose and 75.35 g/L of maltose. No lactic acid or ethanol was detected.

No significant differences were found between the beer produced with the two yeasts in terms of color (EBC 9.83 and 9.73 for *L. thermotolerans* and *L. quebecensis*, respectively) and bitterness (30.19 and 32.05, respectively). Differences were observed for the °Plato that reached 6.33 for *L. thermotolerans*, while *L. quebecensis* resulted in 7.19. No glucose was detected in any of the beers, while some residual maltose was detected, with 2.81 g/L for the *L. quebecensis* fermentation.

As mentioned before, an important ability of the *Lachancea* genera is the ability to produce lactic acid and alcoholic fermentation. However, lactic acid production is strain dependent. For example, the commercial starter Concerto was able to produce about 1.83 g/L of the organic acid [46], while the isolate studied by Domizio et al. (2016) was able to produce 0.25 g/L [16]. However, Zdaniewicz (2020) was barely able to produce it (0.01 g/L) [17]. Kandylis et al. (2023) studied different *L. thermotolerans* strains isolated from wine, in which the higher lactic acid concentration was reported for the 1-7B to isolate that produced 2.4 g/L. While the isolate 1-5B produced 1.6 g/L [18]. Another *Lachancea* strain, *L. fermentati*, has also been studied for brewing. The yeast produced about 1.4 g/L of the organic acid; however, a lower ethanol content was reported (2.5% *v*/*v*) [13]. These results suggest that ethanol production is strain and species-dependent. In our study, the *L. thermotolerans* isolate produced lactic acid concentrations of 1.25 g/L. Similar behavior was observed for the *L. quebecensis* isolate that produced 1.21 g/L, reaching pH values of 4.18 and 4.06, respectively. On the other hand, it is important to note that the medium used for fermentation might also impact the production of the organic acid.

The ethanol content in the produced beer was 3.78 ± 0.49% and 3.50 ± 0.46% (*v*/*v*) for *L. thermotolerans* and *L. quebecensis*, respectively. Domizio et al. (2016) reported higher ethanol values, studying five different *L. thermotolerans* strains and reporting ethanol values higher than 5.5% (*v*/*v*). In contrast, the isolate studied by Zdaniewscz et al. (2020) produced about 4% (*v*/*v*), indicating that ethanol production is strain-dependent. More recently, Kandylis et al. (2023) reported that ethanol production in *L. thermotolerans* is also strain-dependent and might vary between 3.58 to 5.57% (*v*/*v*). While the isolates studied by Bellut et al. (2020) showed ethanol concentrations close to the ones reported here [2.96 to 3.73% (*v*/*v*)]. Considering that low-alcohol beers are defined as those with an ethanol content of 3.5% (*v*/*v*), the isolates studied here could be suitable candidates for reduced-alcohol beer production.

To our knowledge, *L. quebecensis* has not yet been studied as a potential starter culture for beer brewing. Nevertheless, the similar metabolic behavior observed for this isolate, compared to other *Lachancea* strains, suggests it might be a suitable culture for producing low ethanol and sour beer.

### 3.5. Beer Aroma and Sensory Attributes

An important attribute of beers is the perceived aroma. Seven volatiles were determined in the final beers (Table 5). Significant differences were observed for almost all compounds, standing out higher values for ethyl acetate (34.86 ppm), isoamyl alcohol (88.76 ppm), and isoamyl butanol (3.18 ppm) achieved in the fermentation with *L. quebecensis*. On the other hand, the same fermentation showed lower values for dimethyl sulfur (47.19 ppm), 1-propanol (21.63 ppm), and isobutanol (26.39 ppm). According to the odor threshold, in the beer produced with *L. quebecensis*, acetaldehyde, DMS, ethyl acetate, and isoamyl acetate could be perceived by the consumer. This suggests that the beer produced with the *quebecensis* isolate is complex in terms of aroma, standing out fruity aromas such as green apple (acetaldehyde), banana/pear (ethyl acetate), fruity (isoamyl acetate), and vegetables (DMS). Similar to our results, *L. thermotolerans* has been reported before to influence the volatile profile in beer, enhancing the fruity characteristics [17,18,46].

**Figure 3:**
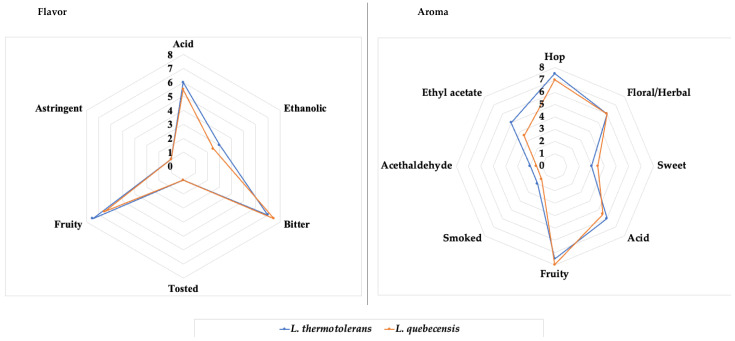
The aroma and flavor profile is perceived by the sensory panel for the two elaborated beers.

The volatile composition agrees with the sensory analysis; the panelist described the beer with a fruity aroma and flavor (Figure 3). According to the judges, the aroma of beer produced with the *L. quebecensis* isolate was evaluated as rich in hop, ethyl acetate, fruity, and floral/herbal. At the same time, the flavor was characterized by its fruit, slightly alcoholic and bitter (Figure 3). The judges scored moderate acid aroma and flavor.

To influence the aroma of flavor, an enzymatic treatment can be used to hydrolyze the malt proteins or adjust the initial FAN to increase nitrogen availability. Nitrogen is a primary source for the production of higher alcohols. However, the results of this study show that an increase in these volatile compounds can be achieved without the need to modify the brewing process or use enzymes but rather by using unconventional yeasts, such as *L. quebecensis*, which fermentation resulted in higher isoamyl alcohol, which was traduced to fruity aroma and flavor.

The sensory panel gave the highest scores to the fruity and bitter aroma attributes. As for flavor, higher scores were given for hop and fruity. Both acid aroma and flavor were also scored with significant values. A slight ethanolic flavor was perceived by the judges (Figure 3).

## 4. Conclusions

Yeast with fermentative potential can be isolated from different environments. In this case, we characterized two *Lanchancea* isolates that showed technological potential for producing craft beer. The beers produced by the yeast isolates resulted in low ethanol concentrations, low pH, and moderate lactic acid concentrations. The beer made by the *L. quebecensis* isolate was characterized as fruity, slightly ethanolic, and moderately acidic. Our results suggest that similar to *L. thermotolerans*, previously reported as able to produce lactic acid, the *quebecensis* isolate, which also shows this ability, can be a potential culture for producing sour beer. Currently, the production of sour beer relies upon the production of organic acids in a secondary spontaneous, and sometimes inoculated, fermentation driven by lactic acid bacteria. The potential of using a yeast culture that results in the same attributes is relevant regarding process control and efficiency. To our knowledge, this is the first time a *L. quebecensis* has been reported as a potential candidate for beer making.

## Figures and Tables

**Table 1:** Yeast species isolated from the studied insects.

Insect	Yeast Identification (Number of Colonies)
Ladybug	*L. thermotolerans* (4)
Corn weevil	*C. boidinii* (1)
	*L. quebecensis* (1)
	*L. thermotolerans* (2)
	*Yarrowia sp.* (1)
	*T. delbrueckii* (1)
Wheat flour weevil	*T. delbrueckii* (1)
	*C. boidinii* (1)
	*P. guilliermondii* (1)
	*L. thermotolerans* (3)
Cabbage worm	*L. thermotolerans* (2)

Identification was done based on the *26S* rRNA sequencing. Numbers in brackets indicate the number isolates.

**Table 2:** The fermentative ability of the non-*Saccharomyces* yeast isolates in a defined glucose medium after seven days of fermentation.

Yeast	CFU/mL	Ethanol	pH	Yield (g Etanol/g Glucosa)	Classification According to Ethanol Yield *
*L. thermotolerans* **	3.82 × 10^8^	3.25 ± 1.77	3.67 ± 0.01	0.17	Very low
L. quebecensis **	1.25 × 10^9^	4.25 ± 1.06	3.72 ± 0.00	0.22	Low
*L. thermotolerans*	1.04 × 10^9^	4.50 ± 0.00	3.69 ± 0.01	0.24	
*L. thermotoleran*s	3.46 × 10^9^	5.25 ± 1.06	3.76 ± 0.04	0.27	
*L. thermotolerans*	8.93 × 10^7^	5.50 ± 0.71	3.65 ± 0.02	0.29	
*L. thermotolerans*	2.08 × 10^8^	5.50 ± 0.71	3.95 ± 0.08	0.29	
*L. thermotolerans* **	2.00 × 10^7^	6.88 ± 2.25	4.51 ± 0.23	0.36	Moderate
*L. thermotolerans*	4.00 × 10^7^	7.00 ± 0.82	4.45 ± 0.25	0.37	
*L. thermotolerans*	3.56 × 10^8^	7.50 ± 0.71	3.66 ± 0.01	0.39	
*L. thermotolerans*	3.00 × 10^6^	7.50 ± 1.29	3.86 ± 0.01	0.39	
*L. thermotolerans* **	2.07 × 10^9^	9.25 ±0.35	3.80 ± 0.00	0.48	Similar to control
*L. thermotoleran*s	2.00 × 10^7^	9.63± 2.36	3.87 ± 0.06	0.5	
*S. cerevisiae* (control)	9.00 × 10^7^	9.52 ± 3.20	4.35 ± 0.39	0.5	Control

Values represent the average of three replicates and two separate runs ± standard deviation. * Classification was done in four categories relative to the yield observed for the control yeast (*S. cerevisiae*). ** Isolates selected for further experimentation.

**Table 3:** Fermentative profile of selected non-*Saccharomyces* yeast isolates in maltose-defined medium.

Yeast	log CFU/mL	Ethanol% (*v*/*v*)	pH
*S. cerevisiae* (control)	9.41	5.50 ± 0.32	5.02 ± 0.29
*L. thermotolerans*	9.36	5.67 ± 0.82	3.16 ± 0.03
*L. thermotolerans*	8.15	4.92 ± 0.36	4.12 ± 0.18
*L. quebecensis* (OP923903)	9.17	4.25 ± 0.45	3.07 ± 0.14
*L. thermotolerans* (OP923897)	9.00	4.50 ± 0.55	5.14 ± 0.49

Values represent the average of three replicates and two separate runs ± standard deviation.

**Table 4:** Physiochemical parameters of the craft beer obtained after 24 days.

Parameters	Initial Values	*L. thermotolerans*	*L. quebecencis*
Color	9.71 ± 1.01	9.83 ± 1.545 ^a^	9.73 ± 1.35 ^a^
Bitterness	58.7 ± 4.85	30.2 ± 4.72 ^a^	32.0 ± 3.06 ^a^
Final Extract (%*w*/*w*)	13.0 ± 0.78	6.3 ± 0.17 ^a^	7.19 ± 1.31 ^b^
Original extract (%*w*/*w*)	12.9 ± 0.76	12.5 ± 0.738 ^a^	12.1 ± 0.604 ^a^
pH	5.55 ± 0.01	4.18 ± 0.11 ^a^	4.06 ± 0.20 ^b^
Ethanol (%*v*/*v*)	ND	3.78 ± 0.49 ^a^	3.50 ± 0.46 ^b^
Lactic acid (g/L)	ND	1.25 ± 0.03 ^a^	1.21 ± 0.05 ^a^
Glucose (g/L)	11.9 ± 1.56	ND	ND
Maltose (g/L)	75.3 ± 1.23	2.75 ± 0.35 ^a^	2.81 ± 0.50 ^a^
FAN (ppm)	185.8 ± 5.27	-	-

Values represent the average ± standard deviation of three replicates. Lowercase letter within columns represent significant differences (*p* < 0.05). ND: Not detected. FAN: Free Amino Nitrogen and Color were determined following the methodology proposed by the European Brewery Convention (EBC).

**Table 5:** The volatile composition of craft beers elaborated with *L. thermotolerans* and *L. quebecensis*.

Volatile Composition (ppm)	*L. thermotolerans*	*L. quebecensis*	Odor Threshold (ppm)
Acetaldehyde	16.9 ± 3.69 ^a^	14.9 ± 8.49 ^a^	20–10
Dimethyl sulfur (DMS)	65.3 ± 18.99 ^a^	47.2 ± 24.71 ^b^	25–50
Ethyl acetate	7.87 ± 1.06 ^a^	34.9 ± 11.87 ^b^	20–40
1-propanol	26.5 ± 5.15 ^a^	21.6 ± 1.70 ^b^	>700
Isobutanol	67.9 ± 10.91 ^a^	26.4 ± 2.04 ^b^	>200
Isoamyl acetate	0.29 ± 0.05 ^a^	3.18 ± 1.37 ^b^	NA
Isoamyl alcohol	74.3 ± 7.55 ^a^	88.8 ± 6.78 ^b^	>70

Values represent the average ± standard deviation of three replicates. Lowercase letter within columns represent significant differences (*p* < 0.05). NA: Not available.

## Data Availability

The data presented in this study are available on request from the corresponding author.
